# Genome-wide identification and comparative analysis of diacylglycerol kinase (*DGK*) gene family and their expression profiling in *Brassica napus* under abiotic stress

**DOI:** 10.1186/s12870-020-02691-y

**Published:** 2020-10-15

**Authors:** Fang Tang, Zhongchun Xiao, Fujun Sun, Shulin Shen, Si Chen, Rui Chen, Meichen Zhu, Qianwei Zhang, Hai Du, Kun Lu, Jiana Li, Cunmin Qu

**Affiliations:** 1 Chongqing Engineering Research Center for Rapeseed, College of Agronomy and Biotechnology, Southwest University, Chongqing, 400716 China; 2 Academy of Agricultural Sciences, Southwest University, Chongqing, 400715 China

**Keywords:** *Brassica*, Diacylglycerol kinase, Gene family, Phylogenetic analysis, Expression profile

## Abstract

**Background:**

Diacylglycerol kinases (DGKs) are signaling enzymes that play pivotal roles in response to abiotic and biotic stresses by phosphorylating diacylglycerol (DAG) to form phosphatidic acid (PA). However, no comprehensive analysis of the *DGK* gene family had previously been reported in *B. napus* and its diploid progenitors (*B. rapa* and *B. oleracea*).

**Results:**

In present study, we identified 21, 10, and 11 *DGK* genes from *B. napus*, *B. rapa*, and *B. oleracea*, respectively, which all contained conserved catalytic domain and were further divided into three clusters. Molecular evolutionary analysis showed that speciation and whole-genome triplication (WGT) was critical for the divergence of duplicated *DGK* genes. RNA-seq transcriptome data revealed that, with the exception of *BnaDGK4* and *BnaDGK6*, *BnaDGK* genes have divergent expression patterns in most tissues. Furthermore, some *DGK* genes were upregulated or downregulated in response to hormone treatment and metal ion (arsenic and cadmium) stress. Quantitative real-time PCR analysis revealed that different *BnaDGK* genes contribute to seed oil content.

**Conclusions:**

Together, our results indicate that *DGK* genes have diverse roles in plant growth and development, hormone response, and metal ion stress, and in determining seed oil content, and lay a foundation for further elucidating the roles of *DGKs* in *Brassica* species.

## Background

In plants, important signaling molecules such as phosphatidylinositol lipids phosphatidic acid (PA), diacylglycerol (DAG), and some lysophospholipids can be activated by distinct environmental stresses, triggering signal transduction cascades and endowing plants with stress resistance [1, 2]. Among these molecules, PA is a key second messenger in the responses to various stresses. PA levels are typically low, accounting for 0.67% of total phospholipids, and PA formation depends on the perception of extracellular stimuli [3], such as drought [4], salinity [5], chilling damage [6], osmotic pressure [7], wounding [8], and pathogen attack [9], or of phytohormones such as ethylene [8], abscisic acid (ABA) [10], brassinolide (BR) [11], and gibberellic acid (GA) [12, 13]. Plants’ ability to overcome such stress events depends on the role of PA in signal perception and transduction [2, 14, 15]. PA is a precursor to all phosphoglycerolipids and its bioaccumulation is pivotal for lipid metabolic flux and membrane composition [16]. PA is generated in the plasma membrane by two different biosynthetic pathways in eukaryotic cells. In the first pathway, phospholipase D (PLD) hydrolyzes phosphatidylcholine (PC) and phosphatidylethanolamine (PE) to generate PA directly. In the second pathway, phospholipase C (PLC) hydrolyzes phosphatidylinositol-4,5-bisphosphate to produce DAG, which is in turn phosphorylated by diacylglycerol kinase (DGK) to yield PA [16].

Previous studies have demonstrated that PA interacts with ABI1 phosphatase 2C, thus promoting ABA signaling in *A. thaliana* [10]. *PLDα* gene expression, protein levels, and enzymatic activity all increase in *A. thaliana* when the leaves are treated with ethylene [8]. In *B. napus* roots, exogenous application of 24-epibrassinolide (EBL) affects PA synthesis through the PLC/DGK pathway under optimal salinity conditions [17]. Furthermore, *DGK* genes are rapidly but transiently regulated in different plant tissues in response to beneficial elements and other ions, including silver (Ag), aluminum (Al), arsenic (As), cadmium (Cd), chromium (Cr), mercury (Hg), and sodium (Na) [18]. Notably, *DGK* genes may be candidates for influencing seed oil content in *B. napus*, according to recent unpublished data from our laboratory. Additionally, diacylglycerol transferase (DGAT) catalyzes DAG to generate triacylglycerol (TAG), which is a major component of vegetable oils in oilseed crops [19]. Therefore, the relative flux of TAG synthesis from de-novo-synthesized or PC-derived DAG can greatly affect the final seed oil content. The *DGK* gene family has been widely characterized in the context of plant stress tolerance, including tomato (*Solanum lycopersicum*, *SlDGK*) [20], *Arabidopsis thaliana* (*AtDGK*) [21], maize (*Zea mays*, *ZmDGK*) [22], rice (*Oryza sativa*, *OsDGK*) [23], apple (*Malus domestica*, *MdDGK*) [4], and soybean (*Glycine max*, *GmDGK*) [24]. However, the genomic analysis of the *DGK* gene family in *Brassica* species has not been reported.

*Brassica napus* (AACC, 2n = 38), a typical allotetraploid of the *Brassica* genus, is an important oil crop planted worldwide, which originated from the hybridization and polyploidization of *B. oleracea* (CC, 2n = 18) and *B. rapa* (AA, 2n = 20) [25]*.* Whole-genome sequences of *B. rapa*, *B. oleracea,* and *B. napus* have recently been assembled [25–27], providing valuable resources for studying the *DGK* gene family in *Brassica* species*.* In this study, we identified *DGKs* in the *Brassica* species and investigated their gene structures, conserved domains, protein properties, evolution, and cis-acting elements through bioinformatics analysis. We also evaluated the expression patterns of *BnaDGKs* in different tissues and their responses to hormone treatment and metal ion induction. Additionally, we studied *BnaDGK* expression patterns in two *B. napus* cultivars with different seed oil contents. This genome-wide identification of *DGK* gene family members in three *Brassica* species provides strong evidence of functional homologies among these *DGK* genes in *Brassica* species*.*

## Results

### Identification and chromosomal distribution of *DGK* genes in *B. napus*, *B. rapa*, and *B. oleracea*

We identified 21, 10, and 11 *DGK* genes in *B. napus*, *B. rapa*, and *B. oleracea*, respectively. *B. napus* had the same number of *DGK* genes as *B. rapa* and *B. oleracea* combined, indicating that *DGK* genes may not have experienced a gene-loss event during polyploidization. In addition, the *B. napus DGK* proteins (*BnaDGKs*) were between 450 and 720 amino acids (aa) in length, corresponding to coding sequence (CDS) lengths of 1353 to 2163 bp; their molecular weights ranged from 49.93 kDa to 79.30 kDa; and their pIs from 5.88 to 8.93 (Additional file 8: Table S1).

The number of exons in the *DGK* genes varied from 7 to 15, with the maximum in *BnaDGK6–2* (Additional file 8: Table S1). Analysis of the physical and chemical characteristics of 21 *DGK* proteins in *B. rapa* and *B. oleracea* revealed that, upon calculation, the average values were approximately equal in *B. napus* and its diploid progenitors.

In plants, previous studies were conducted on the subcellular localization of *DGKs* to the nucleus, plasma membrane, cytoskeleton, and chloroplast [28, 29]. In present study, the subcellular location of all *DGKs* was predicted using the PSORT website (Additional file 8: Table S1). Results showed that most members of cluster 1 were located in the endoplasmic reticulum, except *BnaDGK1–2*, *BnaDGK2–1*, *BolDGK1–2*, and *BolDGK2–2* in the nucleus, in accordance with that *AtDGK1* and *AtDGK2* located in endoplasmic reticulum membranes possess amino-terminal hydrophobic segments that are sufficient to target proteins to the cell membrane to play their role in many signal transduction processes [30]; the members of cluster 2 were located in different parts, for example, most of *DGK3* and *DGK7* were located in the peroxisome and *DGK4s* were localized in the chloroplast, while *BnaDGK3–3* and *BolDGK3–2* occurred in the mitochondrion; while *DGK5* and *DGK6* in cluster 3 were predicted to be located in the peroxisome and cytoplasm, respectively (Additional file 8: Table S1). These subcellular localization results were in accordance with the published report [24] and suggested that *DGKs* have been widely conserved in the same clusters. Furthermore, the diverse localization of plant *DGKs* implies that they might be actively involved in different cellular processes during development and abiotic stress.

Additionally, we investigated the chromosomal localization of *DGK* genes in *Brassica* species based on their physical position in the GFF3 files. In total, 20 of the 21 full-length *DGKs* of *B. napus* mapped to the assembled 11 chromosomes (9 in An genomes and 11 in Cn genomes), while only one gene was assigned to Ann (random A-genome chromosome). In *B. oleracea*, 7 of the 11 full-length *DGKs* were distributed on 5 chromosomes, with the remaining 4 genes randomly located on 4 scaffolds. In *B. rapa*, all full-length *DGKs* were positioned on 6 of the 10 assembled chromosomes (Additional file 1: Fig. S1). Notably, chromosomes An03, An09, Cn03, Ar03, and Ar09 contained the maximum number of genes, i.e., three, whereas chromosomes Cn02, Cn07, Cn09, Co03, and Co07 each contained two genes. This result suggests that *DGKs* have uneven distributions in different species. Statistical analysis showed that seven gene pairs have maintained their relative positions between the An subgenome of *B. napus* and the Ar subgenome of *B. rapa*, and six gene pairs have maintained their relative positions between the Cn subgenome of *B. napus* and the Co genome of *B. oleracea* (Additional file 1: Fig. S1).

### Multiple sequence alignments and sequence characterization of the *DGK* genes

To explore the structure of DGK protein sequences, we performed multiple sequence alignments using ClustalX and visualized the results with Jalview and DOG 2.0. The sequences of two diacylglycerol/phorbol ester (DAG/PE)-binding domains (C1 domain, PF00130; Additional file 2: Fig. S2) indicate that the first and second DAG/PE-binding domains harbor the sequences HX_14_CX_2_CX_19 ~ 22_CX_2_CX_4_HX_2_CX_7_C and HX_18_CX_2_CX_16_CX_2_CX_4_HX_2_CX_11_C, respectively. The two extremely conserved C_6_/H_2_ cores were also observed in cluster 1 *DGKs* of other plants, such as soybean [24], apple [4], and maize [22]. Moreover, the alignment revealed that, except for the orthologs of *AtDGK2*, almost all *DGKs* possess a presumed ATP-binding motif with a GXGXXG consensus sequence in their catalytic domain (DGKc), where G represents glycine and X represents any other amino acid (Additional file 3: Fig. S3). Furthermore, in the homologs of *AtDGK2*, glycine (G) is replaced by alanine (A), but this change does not render the enzyme inactive [21]. Interestingly, *BnaDGK2–1*, with the shortest protein sequence in cluster 1, lacked the C_6_/H_2_ cores, upstream basic region, and an extended cysteine-rich (extCRD-like) domain; it seems probable that *BnaDGK2–1* lost these domains during evolution.

A schematic diagram of the functional domains of cluster 1 DGK proteins drawn using DOG 2.0 showed that compared to the other two clusters, two DAG/PE binding domains (C1), the upstream basic region, and a conserved 15-amino-acid extension were specific to cluster 1 (Additional file 4: Fig. S4). In the upstream basic region, YT and VP residues remained as front and back boundaries, respectively, except in homologs of *AtDGK1*, in which VP was replaced with TP. Furthermore, the second DAG/PE-binding domain included a preserved 15-aa extension, an extCRD-like domain; whereas the combination of the extCRD-like domain and DGKc domain is indispensable to the function of the functionally active DGKs [31]. Overall, the *DGKs* of cluster 1 in *A. thaliana*, rice, apple, soybean, and *B. napus*, as well as its two diploid progenitors have a universal framework: (YT-upstream basic region-VP/TP) – (3 aa) – (DAG/PE-binding domain 1) – (12 aa) – (DAG/PE-binding domain 1/extCRD-like domain) – (~ 130 aa) – (DGKc/DGKa domain). These results suggest that the structure of *DGKs* is extremely conserved in cluster 1.

### Phylogenetic tree and syntenic analysis of the *DGKs* in *B. napus*, *B. rapa*, and *B. oleracea*

**Fig. 1:**
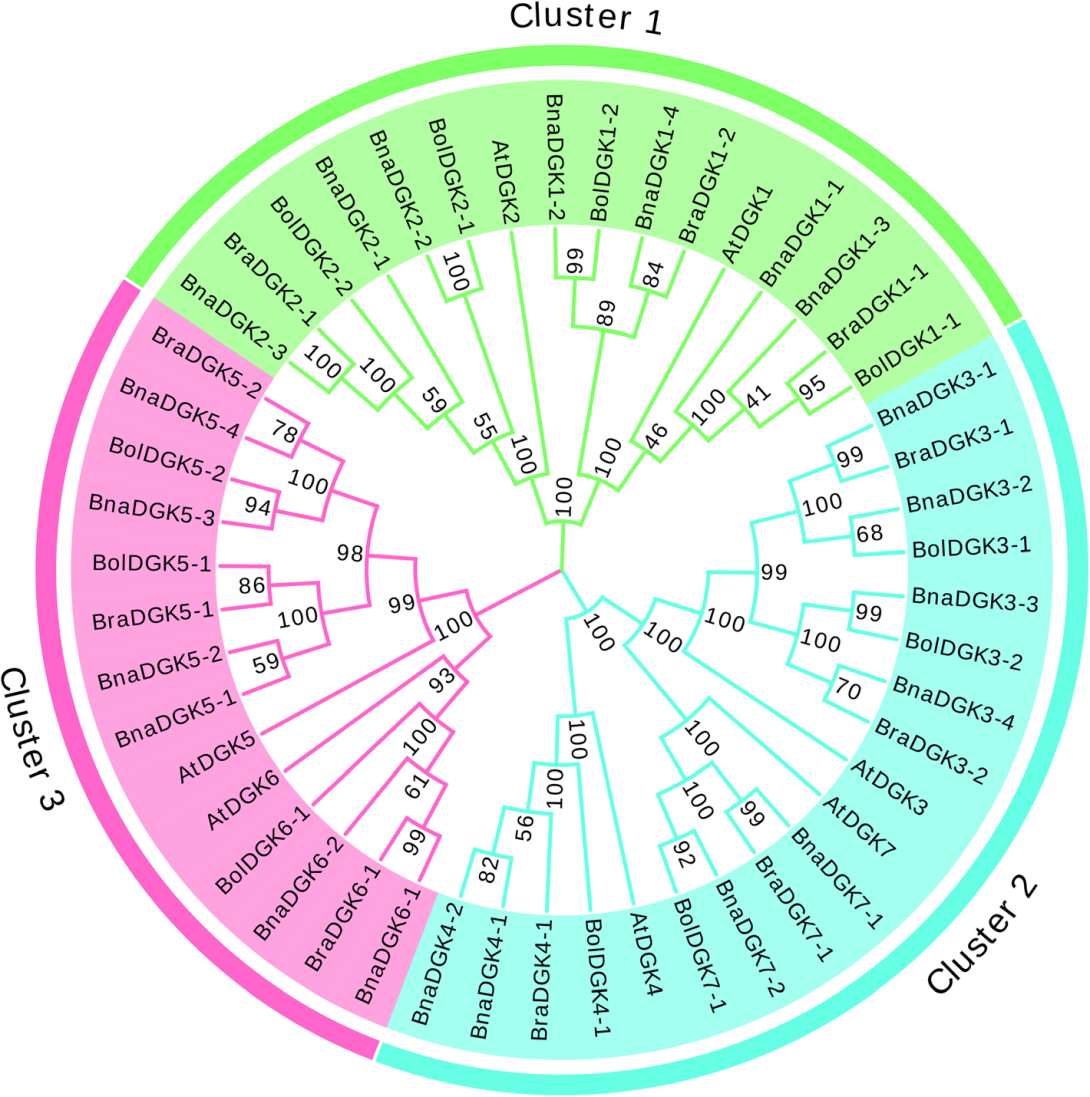
The phylogenetic tree analysis of *DGK* proteins between *A. thaliana* and *Brassica* species. The MEGA7.0.26 was used to do the phylogenetic analysis with the neighbor joining method

To explore the evolutionary relatedness of *DGKs* between *A. thaliana* and *Brassica*, we performed the multiple sequence alignment of *DGKs* using MUSCLE. Subsequently, we constructed a phylogenetic tree based on the DGK protein sequences, including 7, 21, 10, and 11 protein sequences from *A. thaliana*, *B. napus*, *B. rapa*, and *B. oleracea*, respectively (Fig. 1). Generally, the gene number in the *B. rapa*, and *B. oleracea* genome was notably less than three times the *A. thaliana* gene number because some genes might be lost during polyploidy speciation. For example, the *A. thaliana* genes *AtDGK4*, *AtDGK6*, and *AtDGK7* have at most only two homologs in each of the three *Brassica* species. By contrast, the remaining *AtDGK* orthologs possess at least three homologs in *B. napus*. Additionally, the phylogenetic tree is clearly divided into three clusters, designated clusters 1, 2, and 3, in accordance with previous studies in *A. thaliana* [21]. Among them, the *DGK* genes in *B. napus* were closely associated with their corresponding genes in *B. oleracea* and *B. rapa* in each clade, suggesting that these genes might have undergone whole-genome duplication events from diploid parental species (*B. oleracea* and *B. rapa*) to allotetraploid *B. napus*, which had similar functions.

**Fig. 2:**
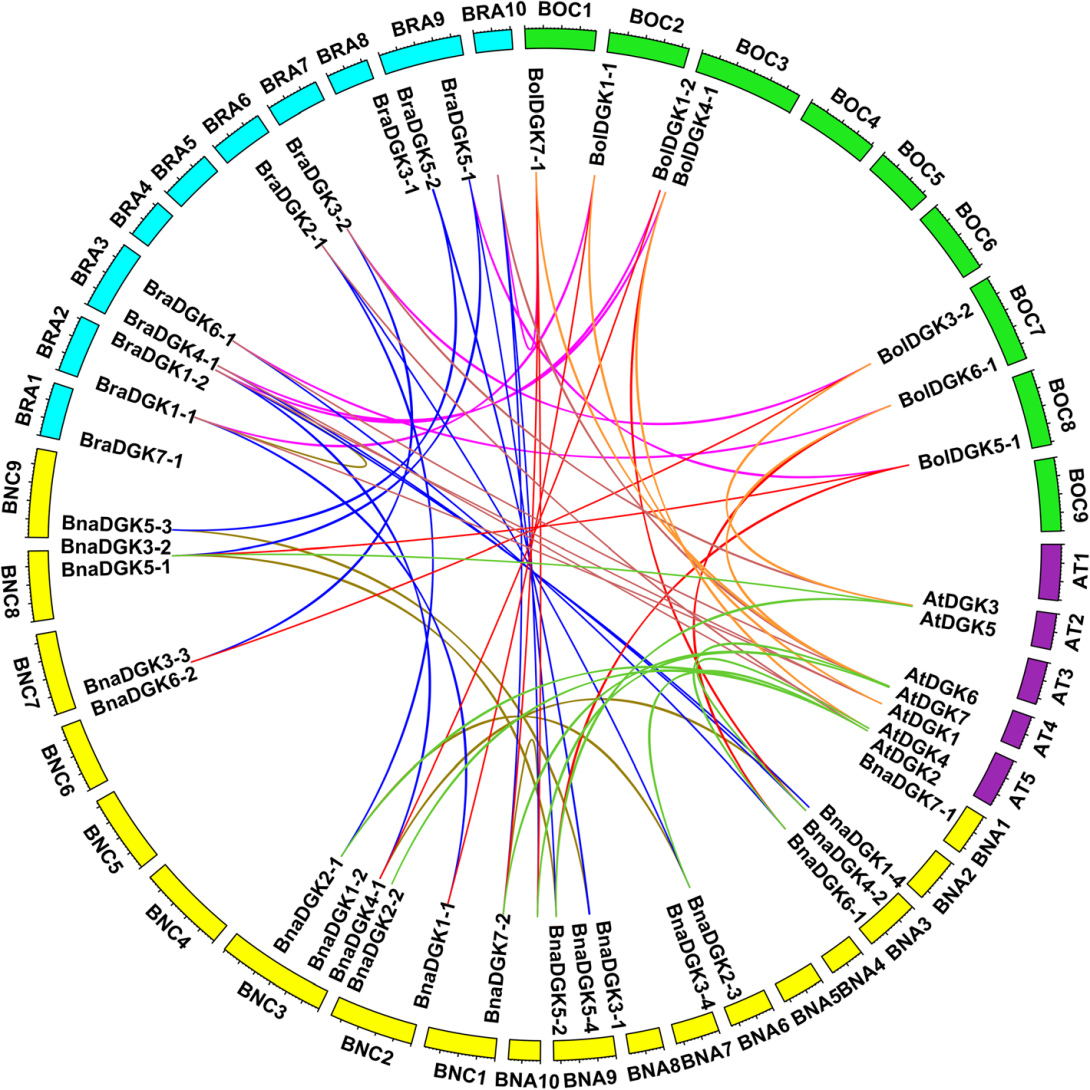
Genome-wide synteny analysis of *DGK* genes from *B. rapa* (Bra), *B. oleracea* (Bol), *B. napus* (Bna), and *A. thaliana* (Atchr). The outer circle indicates the locations of the *AtDGKs*, *BnaDGKs*, *BraDGKs*, and *BolDGKs* on each chromosome. The inner circle indicates the chromosome number of *A. thaliana* (Atchr1–5) in purple, *B. rapa* (BraA1–10) in blue, *B. oleracea* (BolC1–9) in green, *B. napus* (BnaA1–10 and BnaC1–9) in yellow. The syntenic *DGKs* genes were linked using different color lines

We also performed collinearity analysis of *DGKs* in *A. thaliana* and the three *Brassica* species to explore the evolutionary relationship of *DGKs* (Fig. 2). A total of 22 collinear gene pairs between *Brassica* and *A. thaliana* were identified, including 9 AT&Bna gene pairs, 7 AT&Bra gene pairs, and 6 AT&Bol gene pairs (Additional file 8: Table S2). Furthermore, these 21 *DGKs* in *B. napus* were identified as three homologous gene pairs on homologous chromosomes, while three pairs of genes were not located on homologous chromosomes. For example, the gene pair *BnaDGK2–3*& *BnaDGK2–1* was distributed on the An06 and Cn03 chromosomes, respectively, possibly as a result of chromosomal rearrangement and segmental duplication (Additional file 8: Table S2). Our results suggested that the *DGKs* might undergo gene duplication or loss from the diploid parental species (*B. oleracea* and *B. rapa*) to the allotetraploid *B. napus*. Overall, these findings provide the clues for investigating the expansion mechanisms and functional characteristics of *DGK* family genes in *Brassica* species.

### The expansion of *DGK* genes in *Brassica* species

We also studied the *DGK* gene duplication types using MCScanX to determine the expansion patterns of the *DGK* gene family in *Brassica* species. A total of 101,040 genes annotated in the *B. napus* genome [25] were examined in this study, and 21,680 genes (21.46%) were dispersed and 74,035 genes (73.27%) might have undergone segmental duplication. Among them, all identified *DGK* genes belong to segmental duplication except for one dispersed gene *BnaDGK4–1* (Additional file 8: Table S1). Moreover, all *DGK* genes of *B. oleracea* and *B. rapa* were derived from segmental duplication. Our findings showed that segmental duplication appears to play an important role in *DGK* gene expansion in *Brassica* species.

The allopolyploid *B. napus* was formed by hybridization of *B. rapa* and *B. oleracea*, and its estimated formation time was approximately ~ 7500 years ago [25]. To explore the selective pressure on the *DGKs* after duplication events, we calculated the nonsynonymous (*K*_*a*_) and synonymous (*K*_*s*_) substitution rates and the *K*_*a*_/*K*_*s*_ ratio for the 69 identified syntenic gene pairs in the three *Brassica* species and *A. thaliana* (Additional file 8: Table S2). *K*_*a*_/*K*_*s*_ = 1 signifies that genes have experienced neutral selection, whereas *K*_*a*_/*K*_*s*_ > 1 or *K*_*a*_/*K*_*s*_ < 1 indicate that genes have experienced positive or negative selection, respectively. As a result, the *K*_*a*_/*K*_*s*_ values between the *Brassica* species and *A. thaliana* ranged from 0.08 to 0.27. The *K*_*a*_/*K*_*s*_ values for most duplicated *DGK* gene pairs in *B. napus* and each of its diploid progenitors (*B. rapa* and *B. oleracea*) were < 1, except for one gene pair (*BnaDGK7–2*&*BolDGK7–1*) with a value > 1. Furthermore, for three gene pairs (*BnaDGK1–1*&*BolDGK1–1*, *BnaDGK2–1*&*BolDGK2–2*, *BnaDGK7–1*&*BraDGK7–1*), there were no *K*_*a*_/*K*_*s*_ values, because the two genes of each pair had exactly the same CDS. These results showed that most *DGK* genes have undergone purified selection, whereas the gene pair *BnaDGK7–2*&*BolDGK7–1* has been positively selected (Additional file 8: Table S2).

In addition, the divergence time of the duplicated genes was estimated by calculating *K*_*s*_ values. Our estimation showed that the divergence time ranged from 12.11 to 16.75 million years ago (MYA) and averaged 14.43 MYA between the *Brassica* species and *A. thaliana* (Additional file 8: Table S2). This result indicated that the divergence of *Brassica DGK* genes occurred at ~ 14.43 MYA, in accordance with the WGT event between *Brassica* species and *A. thaliana* thought to have occurred approximately 9–15 MYA [32]. For the 6 paralogous gene pairs in *B. napus*, we used *K*_*s*_ values to estimate the time of the whole-genome duplication event. The *K*_*s*_ values ranged from 0.07 to 0.17, with an average of 0.12. The corresponding derived divergence time varied from 2.48 to 5.83 MYA, with an average of 3.84 MYA, which is considerably lower than the average value in *B. napus*, indicating that the divergence of the *DGK* genes in *B. napus* occurred well after the *Brassica* WGT event (Additional file 8: Table S2). This may be because the formation of the *B. napus* species and the *Brassica* WGT event had little effect on the divergence of the syntenic *DGK* genes in *B. napus*.

### Gene structure, domain, and conserved motif analysis

**Fig. 3:**
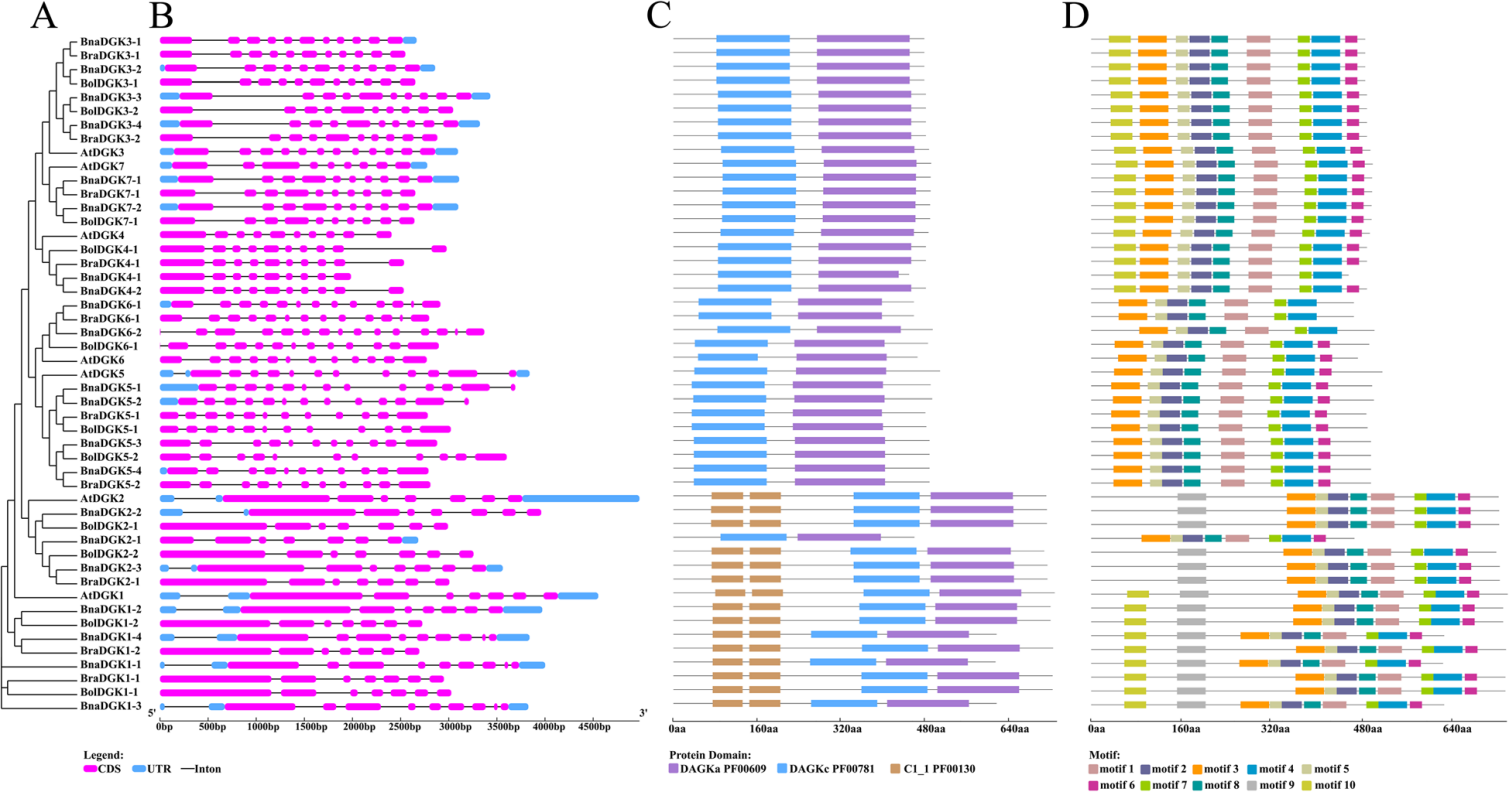
The phylogenetic tree, genestrure, domain location and motif analysis of the identified *DGK* genes in *Brassica* species and *A. thaliana*. **a** The phylogenetic analysis of DGK protein sequences. **b** Exon and intron distribution of the gene structures of DGK family genes. **c** The domain location analysis of DGK protein sequences. **d** The motif compositions of DGK

We studied the intron–exon structures of the *DGK* genes to determine the structural diversity of *DGK* genes in different clusters and to explore whether the gene structure changed during polyploidization (Fig. 3). Each *DGK* cluster possesses a different gene structure. The structure of *DGK* genes from cluster 1 was significantly more conserved than the other two clusters, with an average of 7 exons. Of 16 *DGK* genes in cluster 1, 13 gene pairs had an identical gene structure with the corresponding *A. thaliana* homologs, with 7 exons, whereas 3 genes had 9 exons. By comparison, the number of exons varied from 9 to 12 and from 11 to 15 in clusters 2 and 3, respectively. In cluster 2, 12 out of 19 *DGK* genes had 10 exons. In cluster 3, out of 14 *DGK* genes, 6 genes were composed of 11 exons and 5 genes had 12 exons. In addition, we performed a comparative analysis of 17 gene pairs with the closest genetic distance in the phylogenetic tree, which we considered might have a direct evolutionary relationship. Of these 17 gene pairs, 16 gene pairs had an identical gene structure. Therefore, the *DGK* genes shared a high similarity in exon number during polyploidization.

Furthermore, we analyzed the domain location of DGK proteins to identify the changes in the domain’s position between different clusters or different species (Fig. 3). Although, all the DGK proteins assessed contained a catalytic domain (DGKc, PF00781) and an accessory domain (DGKa, PF00609), only cluster 1 had two C1_1 (PF00130) domains. Furthermore, *BnaDGK2–1* had no C1_1 domain and had the shortest sequence in cluster 1. The C1 (or DAG/PE binding) domain binds an important secondary messenger DAG, as well as the analogous phorbol esters (PE) [33]. Additionally, the positions and lengths of functional domains belonging to the same clusters were generally identical, although they varied between clusters. For instance, most DGKc domains of cluster 1 started from the 261st aa, while the DGKc domains of cluster 2 began at the 82nd aa and the DGKc domains of cluster 3 began at the 35th aa. The location of the DGKa domain was likewise different between the three clusters.

Next, we used the MEME online tool to search for 10 conserved motifs in DGK proteins (Fig. 3 and Additional file 5: Fig. S5). We detected 10 motifs, among which 7 (motifs 1, 2, 3, 4, 5, 7, and 8) occurred in all DGK proteins. In general, DGK proteins in the same cluster displayed parallel motif components. Notably, motif 9 existed only in cluster 1 and constituted the second C1_1 domain of *DGKs*, while motif 10 existed in 28 DGK proteins, including 19 *DGKs* in cluster 2 as well as the *DGKs* homologous to *AtDGK1*. Taken together, *DGK* genes were highly conserved at the protein level among *Brassica* species and *A. thaliana*.

### Expression profiles of DGK genes in different tissues of Brassica species

**Fig. 4:**
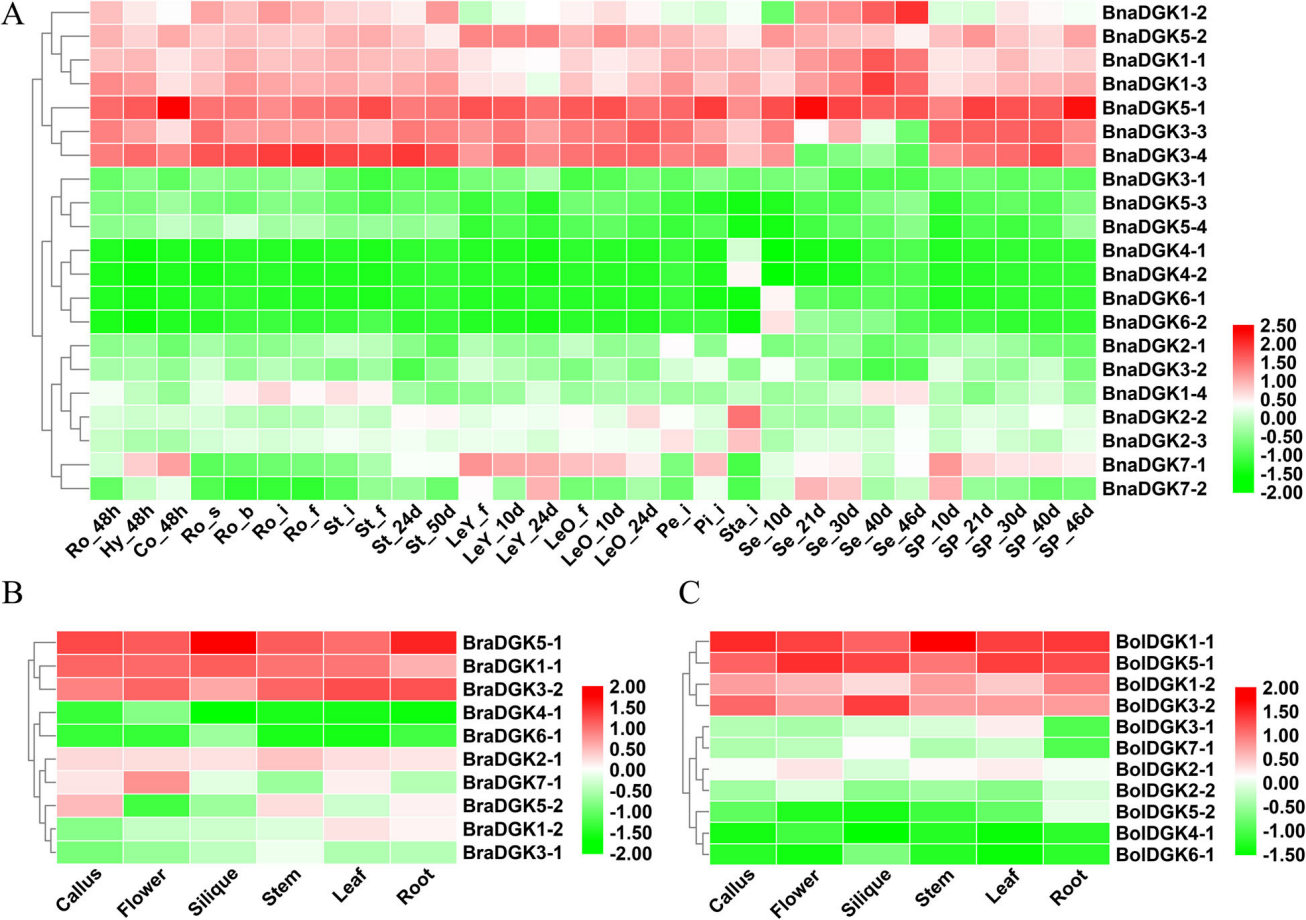
Expression patterns of all *BnaDGKs* in 12 different tissues at different developmental stages in *B. napus* and expression pattern of *BolDGKs* and *BraDGKs* in six tissues. The color bar represents log_2_ expression levels (FPKM)

As we know, the gene functions were predicted by the expression patterns during plant development. To explore the expression patterns of *BnaDGKs*, we analyzed their expression levels in 12 distinct tissues at different developmental stages, including radicle, hypocotyl, cotyledon, root, stem, young leaf, mature leaf, petal, pistil, stamen, seed, and seed pericarp. All samples of 12 tissues at different developmental stages and time points in *B. napus* are detailed in Additional file 8: Table S4. Based on the transcriptome sequencing datasets from *B. napus* ZS11 (the BioProject ID PRJNA358784), we found that 7 *BnaDGKs* were highly expressed in distinct tissues and 7 *BnaDGKs* were expressed in specific tissues, whereas 3 *BnaDGKs* had low expression levels (Fig. 4a). The remaining 4 *BnaDGKs* (*BnaDGK4* and *BnaDGK6*) exhibited almost no expression in most tissues, except that *BnaDGK4* was detected in Sta_i and *BnaDGK6* was detected in Se_10d (Fig. 4a). Several genes were expressed in all tissues except LeY_f, Sta_i, and seed, including *BnaDGK1–1*, *BnaDGK1–2*, *BnaDGK1–3*, *BnaDGK3–3*, *BnaDGK3–4*, *BnaDGK5–1*, and *BnaDGK5–2*. Notably, *BnaDGK2–2* and *BnaDGK2–3* had high expression in Sta_i (Fig. 4a). Our results showed that *DGKs* had relative expression levels in young tissues, in accordance with the previous results [18]. In addition, some genes displayed the tissue-specific expression profiles, for example, *BnaDGK1–4* in stem, *BnaDGK7–1* in leaves and silique pericarps, and *BnaDGK7–2* in silique pericarps, respectively. Overall, *BnaDGKs* had expression detected at different levels in various tissues.

To further study the function of *DGKs*, we then analyzed the expression levels of *DGKs* in six tissues of parental lines *B. rapa* and *B. oleracea* (Fig. 4b, and c). We also observed that the orthologs members of *DGKs* exhibited different tissue-specific expression patterns. For example, the orthologs of *DGK4* and *DGK6* in *B. rapa* and *B. oleracea* displayed the same expression pattern as those of *B. napus*, which had low or almost no expression in all tissues (Fig. 4). This result suggests that they might be pseudogenes or expressed only at specific developmental stages or under special conditions. However, homologs of *DGK1*, *DGK3*, and *DGK5* possessed different expression patterns among them. For example, *BnaDGK5–1* and *BnaDGK5–2* showed markedly higher expression than *BnaDGK5–3* and *BnaDGK5–4*, as did *BolDGK5–1* and *BraDGK5–1* compared to *BolDGK5–2* and *BraDGK5–2*, respectively. The divergent expression patterns can be explained by the pseudogenization and functional divergence.

### Cis-acting elements in the promoters of *DGK* genes

**Fig. 5:**
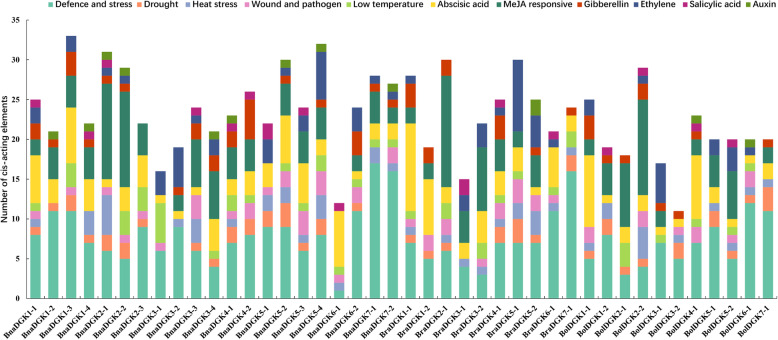
Cis-acting element analysis of *DGK* genes in *B. napus*, *B. oleracea* and *B. rapa*

To explore the regulatory mechanism of *DGK* genes, we carried out an analysis of transcription cis-regulatory elements in the 2000-bp regions upstream of *DGK* gene transcription start sites in three *Brassica* species. We identified and counted cis-acting elements associated with plant development and growth, abiotic and biotic stresses, phytohormone responses and light responsiveness in the promoters of all *DGK* genes (Fig. 5). Of 13 cis-acting elements related to plant development and growth, the as-1 motif (present in 83.3% of the *Brassica DGK* promoters) is involved in the root-specific expression, while the GCN4/AACA motif (28.6%) is involved in endosperm expression (Additional file 8: Table S3).

We also identified four types of abiotic stress elements in the *DGK* promoters: defense and stress responsiveness (MYB/MYC/TC-rich repeats), drought responsiveness (MBS and DRE core), low-temperature responsiveness (LTR), and heat stress responsiveness (STRE) (Fig. 5). Apart from this, two cis-elements related to wounding and pathogen response (W box and WUN motif) were also widely distributed in the *DGK* promoters (Additional file 8: Table S3). For phytohormone-response-related cis-acting regulatory elements, all *DGK* promoters possessed abscisic-acid-responsive element (ABRE). Approximately 83.3 and 71.4% of the *DGK* promoters contained methyljasmonate (Meja)-responsive elements (CGTCA motif and TGACG motif) and gibberellin-responsive elements (GARE-motif, TATC-box, and P-box), respectively, and approximately 66.7% possessed ethylene-responsive element (ERE). Auxin-response elements (AuxRR core and TGA elements) and the salicylic-acid-response element (TCA element) were also present in certain *DGK* gene promoters (Additional file 8: Table S3). Notably, *BraDGK1–1* and *BraDGK4–1* had 11 ABREs and 9 EREs in its promoters, respectively. Moreover, light-responsive regulatory elements consisting of 24 types of different elements were predicted in the *DGK* promoters (Additional file 8: Table S3). These results confirm that the *DGK* genes play a major role in stress resistance and hormone signaling pathways.

### Expression analysis of *DGK* genes in cultivars with different oil contents

**Fig. 6:**
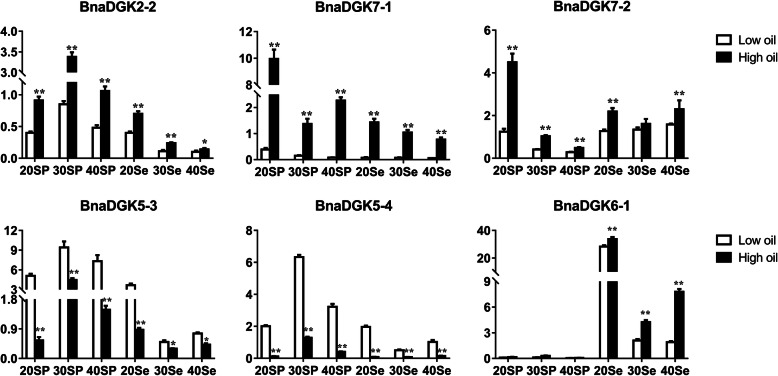
Expression profiles of *BnaDGKs* in the high and low oil content rapeseed cultivars by qRT-PCR. The expression levels of *BnaDGKs* were calculated using 2^−ΔCt^ method. Bar values represent Means ± SEM of three biological replicates with three technical replicates. Asterisks indicate significant differences,* *P* < 0.05, ** *P* < 0.01. Se, Seed; SP, Silique pericarp. The number of days after flower (DAF) is indicated as 20, 30, 40d

DGAT catalyzes the transfer of an acyl chain from a coenzyme A ester to the sn-3 position of sn-1, 2-diacylglycerol to form triacylglycerol [19], while DGKs can catalyze the conversion of DAG to PA. Meanwhile, DGAT and DGK could also compete for the substrate DAG, which is used for the synthesis of TAG and PA, respectively. However, TAG is the main lipid storage form in plants. Therefore, we analyzed the expression patterns of all *BnaDGKs* in samples from two cultivars with different oil contents using qRT-PCR to explore the relationship between *BnaDGKs* and seed oil content. The results showed that most *BnaDGK* genes were differentially expressed in developmental seed and silique pericarp. Except for *BnaDGK6–1* had the highest relative expression in 20 DAF seeds, with a change of up to 33 fold, but it had almost no expression in silique pericarp (Fig. 6). In addition, the relative expression of 8 of 21 *BnaDGK* genes in the seeds, *BnaDGK2–2*, *BnaDGK2–3*, *BnaDGK3–1*, *BnaDGK3–2*, *BnaDGK3–3*, *BnaDGK3–4*, *BnaDGK5–2*, and *BnaDGK7–1*, gradually decreased over time (Fig. 6 and Additional file 6: Figure S6). The results suggest that *DGK* gene expression functions in the early stages of seed development. Importantly, we found that the relative expression levels of *BnaDGK2–2*, *BnaDGK7–1*, and *BnaDGK7–2* had the higher expression levels in development silique pericarp and seeds of high-oil cultivar than that in the low-oil cultivar, but *BnaDGK5–3* and *BnaDGK5–4* had conversely expression patterns (Fig. 6), indicating that they might be involved in the accumulation of seed oil contents. Therefore, these *DGKs* could be selected as excellent candidates for further functional characterization and application in rapeseed breeding programs.

### Expression analysis of *BnaDGKs* under hormone treatment and heavy metal stress

**Fig. 7:**
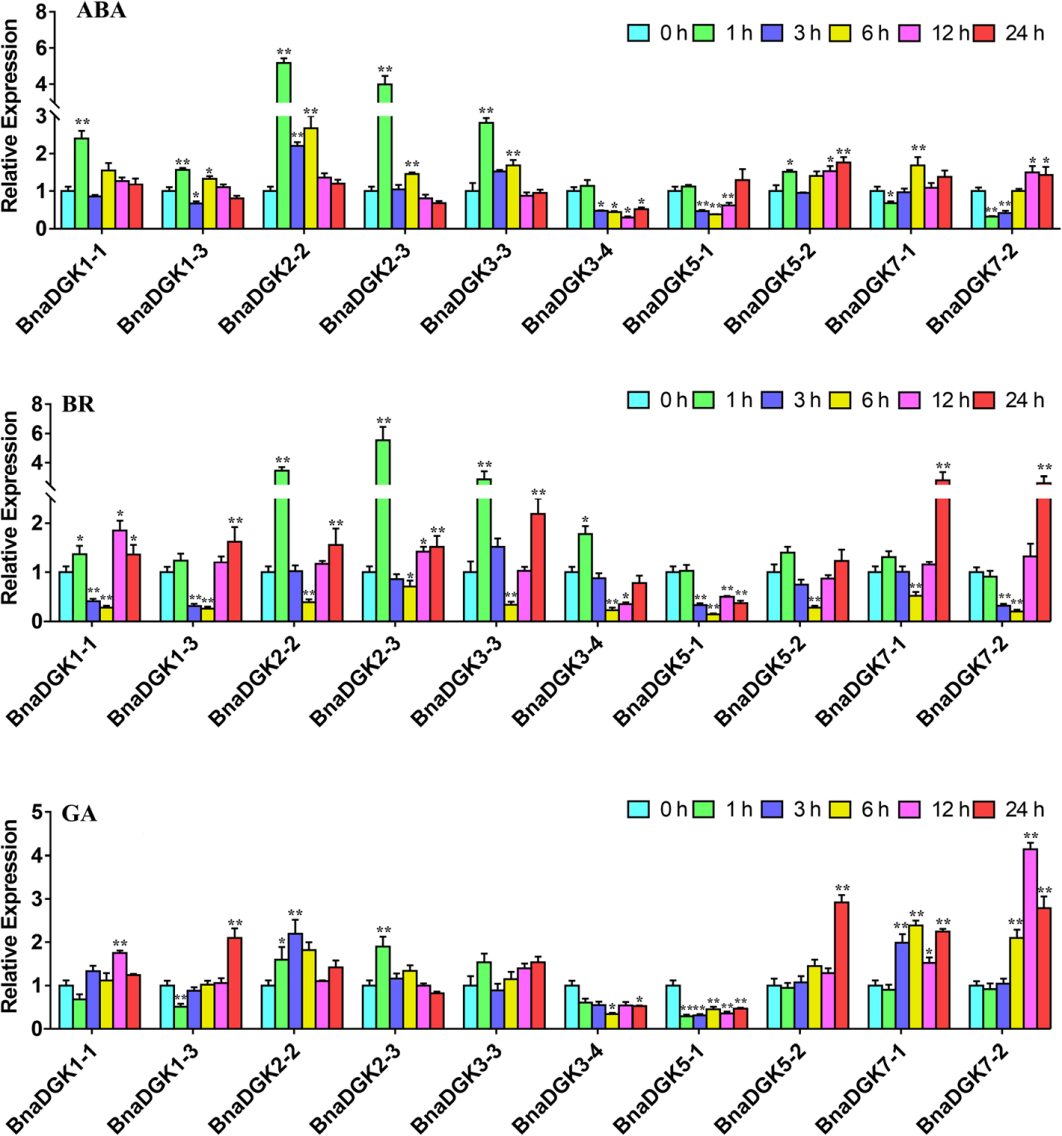
The expression levels of *BnaDGKs* in the leaf under hormone treatments by qRT-PCR. A-C, Expression profiles of *BnaDGKs* under ABA, BR and GA stresses, respectively. The relative expression levels of *BnaDGKs* were calculated using 2^−ΔΔCt^ method and normalized according to the expression values of 0 h (Control) under hormone treatment. Bar values represent Means ± SEM of three biological replicates with three technical replicates. Asterisks indicate significant differences,* *P* < 0.05, ** *P* < 0.01

Based on their expression levels as determined by RNA-seq and the closest genetic distance, five gene pairs were selected and their expression patterns were validated under hormone treatments (ABA, BR, and GA) with qRT-PCR. In general, most of *DGKs* showed a similar expression pattern during the hormone stresses that they showed the sharply up-regulation at 1 h and decreased to the relatively low expression between 3 h and 6 h, then their expression levels were gradually increased after 6 h under ABA and BR treatment (Fig. 7), such as *BnaDGK2–2*, *BnaDGK2–3*, and *BnaDGK3–3*. These results suggested that the gene expression levels of *BnaDGKs* were obviously induced by exogenous hormone and were affected by their own regulation.

For ABA treatment, expression levels of *BnaDGK1–1*, *BnaDGK2–2*, *BnaDGK2–3*, *BnaDGK3–3*, and *BnaDGK5–2* increased to varying degrees at 1 h, by approximately 2.3-, 5.1-, 4.0-, 2.9-, and 1.5-fold, respectively (Fig. 7). Among these, *BnaDGK1–1* and *BnaDGK5–2* possess most ABA response cis-elements, whereas *BnaDGK2–2* the middle and *BnaDGK3–3* the least (Fig. 5). Furthermore, *BnaDGK3–4* and *BnaDGK5–1* expression profiles were significantly downregulated between 3 h and 12 h of ABA exposure, and that of *BnaDGK7–2* was initially downregulated and then gradually increased to peak at 12 and 24 h (Fig. 7). These results suggest that cluster 1 (*BnaDGK1* and *BnaDGK2*) *BnaDGKs* may be responsive to ABA. We only found that the expression pattern of *BnaDGK1* under the ABA treatment was perhaps correlated with the cis-element accumulation (Fig. 5). In terms of BR induction, most *BnaDGK* genes displayed a similar expression pattern. As BR treatment continued, *BnaDGK* expression levels decreased between 1 h and 6 h, and then increased from 6 h to 24 h. In particular, we observed that the expression levels of *BnaDGK2–2*, *BnaDGK2–3*, and *BnaDGK3–3* obviously increased at 1 h, whereas *BnaDGK7–1*and *BnaDGK7–2* exhibited stronger increase at 24 h. The expression levels of most *BnaDGKs* were higher at 24 h than at 0 h, indicating that the BR response mechanism is complex and the role of *DGK* genes is minimal and delayed (Fig. 7). The GA treatment results revealed that except for *BnaDGK1–3*, *BnaDGK3–4*, and *BnaDGK5–1*, the remaining *BnaDGKs* were upregulated with various degrees, with *BnaDGK5–2* and *BnaDGK7–2* exhibiting 2.8-, and 4.0-fold upregulation, respectively. After the GA application, *BnaDGK1–3* expression gradually increased, peaking at 24 h. In addition, *BnaDGK3–4* and *BnaDGK5–1* were downregulated by GA treatment. We found in Fig. 7 that *BnaDGK5–1* lacked GA cis-elements (Fig. 5). Overall, *BnaDGK2–2* and *BnaDGK3–3* were upregulated, while *BnaDGK3–4* and *BnaDGK5–2* were downregulated, in response to the various hormone treatments, implying that some *BnaDGK* genes respond to hormone induction (Fig. 7).

A previous study reported that *DGK* genes in land plants can respond to known beneficial elements as well as to other metal and metalloid ions [18]. We conducted *BnaDGK* expression profiling based on RNA-seq data from *B. napus* cultivars subjected to heavy metal induction (Additional file 7: Fig. S7). Upregulation and downregulation were defined by a log_2_ ratio. The result showed that several *BnaDGKs* were differentially regulated by As^3+^ and Cd^2+^. Thus, induction with As^3+^ resulted in lower expression of *BnaDGK1–4* and *BnaDGK3–2*, but higher expression of *BnaDGK5–4*, *BnaDGK7–1*, and *BnaDGK7–2*. Application of 30 mg/L Cd^2+^ led to the upregulation of *BnaDGK1–1*, *BnaDGK2–2*, *BnaDGK7–2*, and *BnaDGK5–4*, but downregulation of *BnaDGK1–4*. Notably, *BnaDGK1–4* showed some degree of downregulation in response to all metal ion treatments in all cultivars. Our results suggested that some *BnaDGKs* might take part in response to heavy metal stress and their expression might be induced by heavy metal treatments in *B. napus*.

## Discussion

Plants can respond rapidly to environmental stimuli and protect themselves from diverse stressors by activating a series of signal transduction cascades. PA, an emerging signal molecule, mediates signal pathways related to environmental stimuli, and is produced mainly by the PLD and coupled PLC/DGK routes in eukaryotic cells [2]. Moreover, DGK activity has been investigated in several plant species. However, few studies have examined the transcript levels and functions of *DGK* genes. In this work, we identified 21 *BnaDGK* genes in *B. napus*, 10 *BraDGK* genes in *B. rapa,* and 11 *BolDGK* genes in *B. oleracea*. Given that the *A. thaliana* genome encodes 7 *DGK* genes, it is clear that a WGT event has occurred in *Brassica* species since they diverged from *A. thaliana* and that *DGK* genes have been lost since the WGT event [32]. A report that 35% of genes were lost via a deletion mechanism after the *Arabidopsis* and *Brassica* lineages diverged explains why at least 21 *DGKs* are not observed in either *B. rapa* or *B. oleracea* [34]. We identified only 7 collinear gene pairs between *A. thaliana* and *B. rapa*, and 6 collinear gene pairs between *A. thaliana* and *B. oleracea*, perhaps as a result of gene identification or undergo the gene loss during polyploidy speciation. The chromosomal distribution analysis showed that most *DGK* genes in the Ar subgenome of *B. rapa* and the Co subgenome of *B. oleracea* maintained their positions relative to the An and Cn subgenomes of *B. napus*, respectively. Our synteny analysis between *B. napus* and its diploid progenitors indicated that most *DGK* genes in *Brassica* are located in the syntenic regions, with the A subgenome sharing 16 gene pairs and the C subgenome sharing 15 gene pairs.

Polyploidy and WGT are prevalent in the evolutionary history of various species [35]. Most duplicated genes have arisen through whole-genome duplication, and these are often lost or nonfunctional in *Brassica* species [36]. Exploring the functions and characteristics of duplicate genes is important for understanding plant evolution. Our analysis of the *K*_*a*_/*K*_*s*_ values for 69 duplicate genes showed that except for *BnaDGK7–2* and *BolDGK7–1*, all their *K*_*a*_/*K*_*s*_ values were < 1. This implies that these duplicated genes have undergone strong positive selection, which is consistent with previous research indicating that surviving duplicate genes have undergone strong purifying selection [36, 37]. These results indicate that the *DGK* gene family has been well conserved during evolution over time.

Mammalian DGK enzymes are classified into five groups according to sequence homology, whereas plant *DGKs* have been divided into three clusters based on analyses in other plant species [38]. The catalytic region of all plant *DGKs* reported so far consists of a catalytic domain (DGKc, PF00781) followed by an accessory domain (DGKa, PF00609) near the C-terminus, where it may contribute to the normal function of DGKc. The cluster 1 *DGKs* are more complex, with two copies of the DAG-binding domain (C1, PF00130), an upstream basic region, and an extended CRD in their N-termini. The DAG/PE-binding domain features the C_6_/H_2_ cores. By contrast, cluster 2 *DGKs* harbor only the DGKa and DGKc domains, whereas cluster 3 *DGKs* may display an extCRD-like domain generated by alternative splicing [2, 39]. Moreover, since a GXGXXG consensus sequence in their catalytic domain (DGKc) is the ATP-binding motif, *DGKs* can use ATP as an energy generator to catalyze the conversion of DAG to PA. Interestingly, *DGK1p*, a novel *DGK* gene in the yeast *Saccharomyces cerevisiae*, utilizes CTP, rather than the standard ATP, as phosphate donor in forming phosphatidate [40]. The sequence of *S. cerevisiae DGK1p* is unlike that of *DGKs* from other species and contains a CTP transferase domain essential for the protein’s *DGK* activity [40]. The combination of *DGK1p* and phosphatidate phosphatase encoded by *PAH1* can regulate the levels of DAG and PA for phospholipid synthesis [41].

Eukaryotic cells contain two categories of *DGKs*. On the one hand, inactive *DGKs*, stimulated by inositol phosphate metabolic signaling, can catalyze DAG to generate phosphoglycerols. On the other hand, active *DGKs* with greater cellular activity can phosphorylate DAG to yield PA [42]. In eukaryotic species, PA is usually produced from the PLD pathway when plants are subjected to drought [43], oxidative stress, or physical damage [16], whereas PA accumulation via the PLC/DGK pathway is promoted by pathogens and xylanase [16]. Additionally, cold and salt stresses can lead to fast, transient production of PA through both pathways [44]. In *A. thaliana*, transcript expression profiling revealed that *AtDGK1* and *AtDGK2* are implicated in the plant cold response [45]. Furthermore, *AtDGK2* expression can be transiently induced by the wounding signal [46]. A recent study showed that *AtDGK4* is highly expressed in pollen, and a homozygous *AtDGK4* mutation affects not only the male germline but also the vegetative tissue [47]. Overexpression of the rice *DGK* gene *OsBIDK1* can enhance disease resistance in transgenic *N. tabacum* [48], and suppressing *OsDGK* gene expression results in a distinct depletion of transcripts of the defense gene *OsNPR1* and the salt-responsive gene *OsCIPK15* [23]. In addition, transcript levels of *MdDGK4* and *MdDGK8* in apple were induced by salt and drought stress, respectively [4]. In soybean, *GmDGK10* transcripts showed dramatic upregulation in response to PEG stress in root tissue, and *GmDGK8* and *GmDGK9* were significantly upregulated in the presence of NaCl [24]. Additionally, previous studies have indicated that PA is involved via the PLC/DGK route in various hormone pathways, such as the ethylene [8], ABA [10], and BR [17] pathways. Furthermore, the *DGK* gene response to beneficial elements and other ions has been demonstrated [18].

Rapeseed is one of the most widely grown oil crops worldwide, and seed oil content is a crucial agronomic trait in rapeseed breeding. Therefore, exploring ways to increase the oil production of *B. napus* is of great agricultural and economic significance. DAG can be catalyzed by DGAT to form TAG, a major contributor to vegetable oils in oilseed crops. Thus we infer that the *DGK* genes may be candidate genes for improving seed oil content in *B. napus*. In this study, we investigated the effects of *BnaDGK* genes on seed oil content using qRT-PCR analysis. Several *BnaDGK* genes were highly expressed in the sample from the high-oil-content cultivar, whereas *BnaDGK5–3* and *BnaDGK5–4* had distinctly high expression levels in the samples from the low-oil-content cultivar, indicating that differentially expressed *BnaDGK* gene family members should be further functional identified and applied in rapeseed breeding programs.

Nonetheless, transcription level and functional analysis of the response of *DGK* gene family members to hormone treatments and metal stress are fragmentary. In terms of hormone treatments, PA interacts with ABI1 phosphatase 2C, thus promoting ABA signaling in *A. thaliana* [10]. In *B. napus* root, exogenous application of EBL resulted in PA synthesis through the PLC/DGK pathway under optimal salinity conditions [17]. In our study, cluster 1 *BnaDGK* genes (*BnaDGK1* and *BnaDGK2*) may respond to ABA treatment. *BnaDGK2–2*, *BnaDGK3–3*, *BnaDGK3–4*, and *BnaDGK5–2* are potential candidate genes in response to hormone treatments, as *BnaDGK2–2* and *BnaDGK3–3* were upregulated, and *BnaDGK3–4* and *BnaDGK5–2* were downregulated in response to three types of hormone treatments.

Meanwhile, heavy metals, including copper, manganese, cobalt, zinc, and chromium could be toxic to plants, disrupting plant metabolic processes, inhibiting plant growth, or causing plant death [49, 50]. Of these, excessive Cd can produce toxic effects in plants by reducing chlorophyll contents, inhibiting root growth, and perturbing water homeostasis [51]. However, root length, total biomass dry weight, and plant height of *Lonicera japonica* plants increase in the presence of 0.5–5.0 mg/L Cd [52]. The expression levels of all *AtDGK* genes except *AtDGK6* were repressed by Cd, whereas *OsDGK8* expression was slightly upregulated [18]. In our study, *BnaDGK1–1*, *BnaDGK2–2*, and *BnaDGK5–4* were upregulated, while *BnaDGK1–4* was downregulated in *B. napus* exposed to 30 mg/L Cd^2+^. Our results thus contribute further evidence that Cd^2+^ influences *DGK* gene activities.

A previous study has provided direct evidence for As uptake by roots via high-affinity *PHT1* transporters in *A. thaliana* and rice [53]. Furthermore, As stimulates the uptake of Pi, providing some growth benefits at the modest concentrations [54]. As (in the form of NaHAsO4) upregulates *OsDGK2* and downregulates *OsDGK3a* and *OsDGK8* expression in rice [18]. In our study, *BnaDGK1–4* and *BnaDGK3–2* were downregulated, whereas *BnaDGK5–4*, *BnaDGK7–1*, and *BnaDGK7–2* were upregulated, in response to As^3+^ treatment. These observations suggest a possible role of *DGK* genes in As^3+^ absorption. The cis-acting elements were also predicted from the promoter regions of *DGK* genes, and a large number of abiotic and biotic stress elements in these promoters were identified, including MYB/MYC/TC-rich repeats, DRE, LTR, and the W box and WUN motif. Apart from these elements, most *DGK* promotors analyzed contain ABREs, Meja-responsive elements (CGTCA motif and TGACG motif), and gibberellin-responsive elements (GARE-motif, TATC-box, and P-box), as well as EREs.

Together, these results confirm that *DGK* genes play a major role in stress resistance and hormone signaling pathways. Our findings provide useful information on the evolutionary aspects of the *DGK* gene family in *Brassica* genome and assist to reveal the biological functions of *DGKs* in response to both oil metabolism and abiotic stress.

## Conclusions

In this study, we provided a complete overview of the *DGK* gene family in *B. napus* and its diploid progenitors, and analyzed its evolutionary patterns, including gene duplication history and positive selection level. We identified 21, 10, and 11 *DGKs* in *B. napus* and its diploid progenitors *B. rapa* and *B. oleracea*, respectively. We also investigated their chromosome distribution, gene structure, domain, and motif pattern, as well as the presence of cis-regulatory elements. The expression patterns of *DGK* gene family members in diverse tissues at different developmental stages showed that except for those homologous to *AtDGK4* and *AtDGK6*, the *DGK* genes expressed in most tissues. Further, the expression patterns of *DGK* genes under hormone treatment and metal ion induction indicated that some *DGK* genes respond to these treatments. Finally, several *BnaDGKs* expression profiles in cultivars with different seed oil contents were not completely consistent. Together, these results indicate that *DGK* genes have various roles in plant growth and development, hormone response, metal ion stress response, and the control of seed oil content. This work improves our understanding of the evolution of the *DGK* gene family and provides a reference for future studies.

## Methods

### Identification and nomenclature of *DGKs* in *B. napus*, *B. rapa* and *B. oleracea*

Genomic sequences, coding sequences, and protein sequences of *A. thaliana*, *B. napus*, *B. rapa*, and *B. oleracea* were downloaded from the TAIR database (http://www.arabidopsis.org) and the Brassica Database (BRAD, http://brassicadb.org/brad). Seven *AtDGK* sequences from *A. thaliana* were used as queries to identify the candidate *DGK* genes in *Brassica* species via a BLASTp search with a threshold *e*-value of 1*e*-20 [55]. Furthermore, the Pfam database (http://Pfam.sanger.ac.uk/) and the conserved domain database (CDD) of the US National Center for Biotechnology Information (NCBI) (https://www.ncbi.nlm.nih.gov/Structure/cdd) were used to examine two conserved domains (DGKc, PF00781 and DGKa, PF00609) of the putative *DGK* genes. In this study, only proteins containing the complete DGKa and DGKc domains were considered as *DGK* genes.

For nomenclature, a species prefix, such as ‘*Bna*’ for *B. napus*, was used, followed by *DGK* and two numbers. The first number refers to the homologous *Arabidopsis* gene, and the second represents the degree of homology to the corresponding *Arabidopsis* gene, where ‘-1’ represents the highest homology, followed by ‘-2’, and so on: for example *BnaDGK1–1*.

### Multiple sequence alignments and protein properties of all *DGKs* in *B. napus*, *B. rapa*, *B. oleracea*, and *A. thaliana*

The multiple sequence alignments of the *DGK* proteins were established with the ClustalX [56] program, using the default parameter mode. Then, Jalview 2.11.0 [57] and DOG 2.0 [58] software were used to visualize the multiple sequence alignment. In addition, molecular weight (kDa) and isoelectric point (pI) values of each *DGK* protein sequence were predicted using the ExPASy server (http://expasy.org).

### Chromosomal distribution, phylogenetic tree, gene duplication, and syntenic analysis of the *DGKs* in *B. napus*, *B. rapa* and *B. oleracea*

The chromosome location information for the *DGK* genes of *B. napus* and its two diploid progenitors was collected from the BRAD database, and the chromosome distribution was plotted with MapChart 2.0 [59]. To comprehensively understand the evolutionary relationship of the *DGK* gene family members, a phylogenetic tree was constructed with MEGA 7.0.26 using the neighbor-joining (NJ) method [60] among four species (*A. thaliana*, *B. napus*, *B. rapa*, and *B. oleracea*). A series of parameters were as follows: the Poisson model, pairwise deletion, and conserved sequences with coverage of 100% and 1000 bootstrap replicates. Finally, the phylogenetic tree was visualized using the EvolView website (http://www.evolgenius.info/evolview).

To identify forms of gene duplication of *BnaDGKs*, only a total of 101,040 *B. napus* annotated gene sequences were aligned using BLASTp, with an *e*-value of 1*e*-10. Then, the MCScanX software [61] with default parameters was used to classify the duplication patterns of the *DGK* genes, and TBtools software [62] was used to calculate the synonymous (*K*_*s*_) and nonsynonymous (*K*_*a*_) mutation rates of the duplicated *DGK* gene pairs. Divergence time was inferred using the formula T = *K*_*s*_/2R, where *K*_*s*_ represents the synonymous substitutions per site and R is the rate of divergence. For dicotyledonous plants, the assumption R is 1.5 × 10^− 8^ synonymous substitutions per site per year [63]. For the syntenic analysis of *DGKs*, we used Multiple collinear scanning toolkits (MCScanX) to produce the collinearity blocks across the whole genome with the default parameters [61]. Then, the collinearity gene pairs of the *DGK* family were visualized by Advanced Circos programs of TBtools [64].

### Protein domain, motif, and gene structure analysis of all *DGKs* in *B. napus*, *B. rapa*, *B. oleracea*, and *A. thaliana*

To identify conserved domains of *DGKs*, the NCBI conserved domain search (www.ncbi.nlm.nih.gov/Structure/cdd/wrpsb.cgi) was used and the results were confirmed by conducting a Pfam database search. A search for conserved motifs of *DGK* proteins was performed with MEME 5.0.1 (http://meme-suite.org/) using default settings, except that the number of motifs was set to 10. Subsequently, the Gene Structure Display Server (GSDS 2.0, http://gsds.cbi.pku.edu.cn/) was used to conduct an exon–intron structure analysis of *DGK* genes. An integrated schematic including the phylogenetic tree, gene structure, motif, and conserved domain was visualized using EvolView.

### Promoter elements analysis and subcellular localization of all *DGKs* in *B. napus*, *B. rapa*, *B. oleracea*, and *A. thaliana*

A 2000 bp region upstream of the translation start sites of each *DGK* gene was acquired from Brassica database (BRAD) as a promoter sequence, and the cis-acting elements were analyzed using the PlantCARE website (http://bioinformatics.psb.ugent.be/webtools/plantcare/html/). The subcellular localization was predicted using the PSORT online website (http://psort1.hgc.jp/form.html).

### Expression analysis of all *DGKs* in different tissues of *B. napus*, *B. rapa*, and *B. oleracea*

First, total RNA was isolated using the EZ-10 DNAaway RNA Mini-prep Kit (Sangon Biotech Co., Ltd., Shanghai, China) for the rapeseed cultivar Zhongshuang 11 (ZS11) in distinct tissues at different developmental stages. The cDNA library construction and RNA sequencing were performed as described previously [65] at Novogene Bioinformatics Technology (Beijing, China) and were deposited in the BioProject database (BioProject ID PRJNA358784). Then the relative expression values in the bars were calculated based on FPKM values (fragments per kilobase of exon model per million) using Cufflinks with default parameters [66], and expression patterns of the candidate *BnaDGKs* were analyzed at 12 tissues (radicle, hypocotyl, cotyledon, root, stem, young leaf, mature leaf, petal, pistil, stamen, seed, and seed pericarp; Additional file 8: Table S4 and Table S5). Furthermore, six tissues (callus, flower, silique, stem, leaf, and root) RNA-seq data of cultivated *B. oleracea* and *B. rapa* were obtained from the NCBI GEO database (https://www.ncbi.nlm.nih.gov/geo/browse/; accession numbers GSE42891 and GSE43245). All *BnaDGKs*, *BraDGKs*, and *BolDGKs* expression levels were also estimated using FPKM (Additional file 8: Table S5 and Table S6).

In addition, transcriptome sequencing datasets of five rapeseed cultivars under heavy metal stress were generated and analyzed as described above, which were treated cultivars P070, P085, and P087 with 15 mg/L As^3+^ and cultivars P085, P155, and P163 with 30 mg/L Cd^2+^, respectively (Additional file 8: Table S7). Gene expression levels of *BnaDGKs* were estimated using FPKM values. Heatmaps of all *DGKs* were drafted using TBtools [64], which was normalized by Log_2_ (FPKM values).

### Plant materials, hormone treatments, and heavy metal stress

Two *B. napus* cultivars were used in this study, P594 (high oil content, 43%) and P134 (low oil content, 34%), both provided by the Chongqing Rapeseed Engineering Technology Research Center. Both cultivars were grown under natural conditions in Chongqing, China, and inflorescences were bagged before blossoming to prevent pollen contamination. Finally, developing seeds and silique pericarp of two cultivars were collected at 20, 30, and 40 days after flowering (DAF) and preserved at − 80 °C for further analysis.

For hormone treatment, ZS11 seeds were sown and grown in an artificial climatic chamber at 25 °C with a 16 /8 h (day/night) photoperiod. Two-week-old ZS11 seedlings were treated with 50 μM ABA, GA, or BR for 0, 1, 3, 6, 12, and 24 h, respectively, and their leaves were immediately collected. All materials were frozen in liquid nitrogen and stored at − 80 °C until use.

Five rapeseed cultivars (P070, P085, P087, P155, and P163) with extreme phenotypes for heavy metal response (strong of weak resistance) were selected from two hundred rapeseed accessions under different concentrations of two heavy metals (As^3+^ and Cd^2+^). Subsequently, we treated rapeseed cultivars (P070, P085, and P087) with 15 mg/L As^3+^ and rapeseed cultivars (P085, P155, and P163) with 30 mg/L Cd^2+^ as the optimal concentration in this study. In brief, the filled seeds for each accession were sown on the filter papers in the petri dishes (9 × 9 cm), which were treated with distilled water (Control) and 15 mg/L As^3+^ and 30 mg/L Cd^2+^ (Treatment), respectively. The growth conditions were kept at 25 °C with long-day conditions (16 h light/8 h dark, 5000 Lux). After 7 days, whole plants were frozen in liquid nitrogen and stored at − 80 °C until use.

### RNA extraction, complementary DNA synthesis, and quantitative real-time PCR analysis of *BnaDGK* genes

Total RNA was extracted with the EZ-10 DNAaway RNA Mini-prep Kit (Sangon Biotech Co., Ltd., Shanghai, China) according to the manufacturer’s instructions. Subsequently, the gel electrophoresis and a NanoDrop 2000 spectrophotometer were used for detecting the quality and concentration of each RNA sample, and the qualified RNA were used for further analysis. The first-strand cDNA was synthesized from 1 μg RNA by reverse transcription PCR (RT-PCR) with a Perfect Real-Time Synthesis Kit (TaKaRa Biotechnology, Dalian, China). The diluted cDNA after reverse transcription was used as the template for real-time quantitative RT-PCR. Real-time quantitative PCR was conducted with a BIO-RAD CFX96 qPCR instrument and SYBR II (TaKaRa). Each 20 μl PCR mixture that included 10 μl of SYBR® Premix Ex Taq™ II, 2 μl (100 ng) of template cDNA, and 0.4 μM of each PCR primer. The RT-PCR protocol was set to 95 °C for 30 s and 35 cycles of 95 °C for 5 s, followed by 56–60 °C (depending on the primers used) for 30 s. All samples were amplified in three biological replicates and three technical replicates. The 2^−ΔΔCt^ value was used to measure the relative expression levels of *BnDGKs* under hormone treatment [67]; and the 2^−ΔCt^ value was used to measure the relative expression levels of *BnDGKs* between low and high oil content rapeseed [68]. *B. napus ACTIN7* (*BnaACTIN7*, GenBank Accession No. AF024716) was used as the housekeeping gene. All qRT-PCR primers are listed in Additional file 8: Table S8.

### Statistical analysis

The expression levels were defined as mean ± standard error of mean (SEM), and three biological replicates were performed in each experiment. Data was subjected to a one-way ANOVA using SPSS 15.0 (SPSS, Inc., Chicago, Ill) to define the significance differences between the mean values. Differences with *p* values of ≤0.05 and ≤ 0.01 were considered significant and highly significant, respectively.

## Supplementary information

**Additional file 1: Figure S1.** Chromosomal distribution of *DGKs* in *B. napus*, *B. rapa* and *B. oleracea*, the scale bar is showed in the figure.

**Additional file 2: Figure S2.** The multiple sequence alignment of two DAG/PE-binding domain (C1 domain) domains. (A) The first DAG/PE-binding domain, (B) The second DAG/PE-binding domain.

**Additional file 3: Figure S3.** The multiple sequence alignment of each cluster DGKc domain among all *DGK* genes.

**Additional file 4: Figure S4.** A detailed view of domains of *DGK* genes in cluster 1 including two C1 domains, the upstream basic regions and the extended cysteine-rich domain (extCRD).

**Additional file 5: Figure S5.** The detailed information of Motif logos are obtained from the MEME Suite website.

**Additional file 6: Figure S6.** Quantitative RT-PCR analysis of the remaining *BnaDGKs* in different seed oil content materials. The expression levels of *BnaDGKs* were calculated using 2^−ΔCt^ method. Bar values represent Means ± SEM of three biological replicates with three technical replicates. Asterisks indicate significant differences,* *P* < 0.05, ** *P* < 0.01. Se, Seed; SP, Silique pericarp. The number of days after flower (DAF) is indicated as 20, 30, 40d.

**Additional file 7: Figure S7.** The heatmap of *BnaDGKs* expression profiling in response to As and Cd stress. The up-down regulation was defined with log_2_ ratio.

**Additional file 8: Table S1.** Identification of *DGKs* in *B. napus* and its diploid progenitors with their physico-chemical and bio-chemical properties (ER: Endoplasmic reticulum; Nucl: Nucleus; Pero: Peroxisome; Mito: Mitochondria; Chlo: Chloroplast; Cyto: Cytoplasm); **Table S2**. The *K*_*a*_/*K*_*s*_ values and MYA for duplicated *DGK* gene pairs; **Table S3**. The detailed information of cis-acting analysis among *DGKs* in *B. napus*, *B. oleracea*, and *B. rapa*; **Table S4**. *B. napus* ZS11 tissues and organs in this study; **Table S5**. The FPKM values of *DGK* genes in *B. napus* by RNA-Seq analysis; **Table S6**. The FPKM values of *DGK* genes in *B. rapa* and *B. oleracea* by RNA-Seq analysis; **Table S7**. The FPKM values of *DGK* genes in *B. napus* by RNA-Seq analysis under metal ion stress; **Table S8**. Primers used to amplify the *BnaDGK* genes and reference genes using qRT-PCR.

## Data Availability

RNA-seq of *B. napus* variety Zhongshuang 11 (ZS11) in distinct tissues at different developmental stages are available in the NCBI Sequence Read Archive (SRA) database under the accession number PRJNA358784. RNA-seq data for expression profiles of *B. rapa* and *B. oleracea* in different tissues were acquired from NCBI gene expression omnibus (GEO) database (GSE43245 and GSE42891). All other datasets supporting the results of this article are included within the article and its Additional files.

## Abbreviations

*DGK*: Diacylglycerol kinase
*DAG*: Diacylglycerol
*PA*: Phosphatidic acid
*DGKc*: Diacylglycerol kinase catalytic
*DGKa*: Diacylglycerol kinase accessory
*WGT*: Whole-genome triplication
*qRT-PCR*: Quantitative real-time polymerase chain reaction
*ABA*: Abscisic acid
*BR*: Brassinolide
*GA*: Gibberellic acid
*PLC*: Phospholipase C
*PLD*: Phospholipase D
*PC*: Phosphatidylcholine
EBL: 24-epibrassinolide
*As*: Arsenic
*Cd*: Cadmium
*DGAT*: Diacylglycerol transferase
*TAG*: Triacylglycerol
*DAG/PE*: Diacylglycerol/phorbol ester
*Ka*: Nonsynonymous
*Ks*: Synonymous
*MYA*: Million years ago
*ABRE*: Abscisic-acid-responsive element
*ERE*: Ethylene-responsive element
*DAF*: Days after flowering
*NJ*: Neighbor-joining
*FPKM*: Framents per kilobase of exon per million mapped framents
