# Supplementary figures and images for: Genome-wide identification and comparative analysis of diacylglycerol kinase (DGK) gene family and their expression profiling in Brassica napus under abiotic stress
Source: BMC Plant Biol. 2020 Oct 15;20:473. doi: 10.1186/s12870-020-02691-y (PMC7559766; doi:10.1186/s12870-020-02691-y)

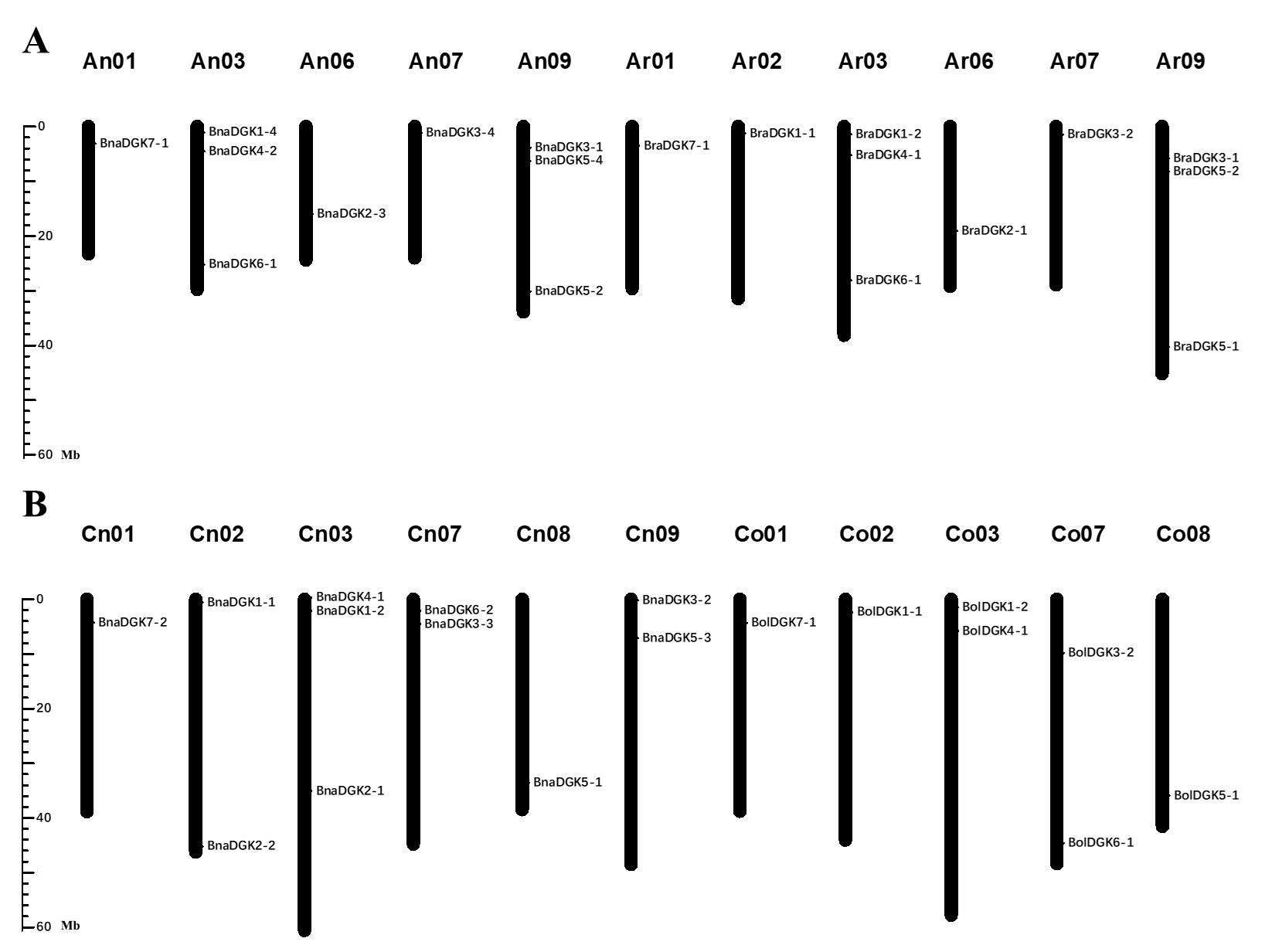

Supplement: Supplementary file 1 — Additional file 1: Figure S1. Chromosomal distribution of DGKs in B. napus, B. rapa and B. oleracea, the scale bar is showed in the figure. [file 12870_2020_2691_MOESM1_ESM.tif]

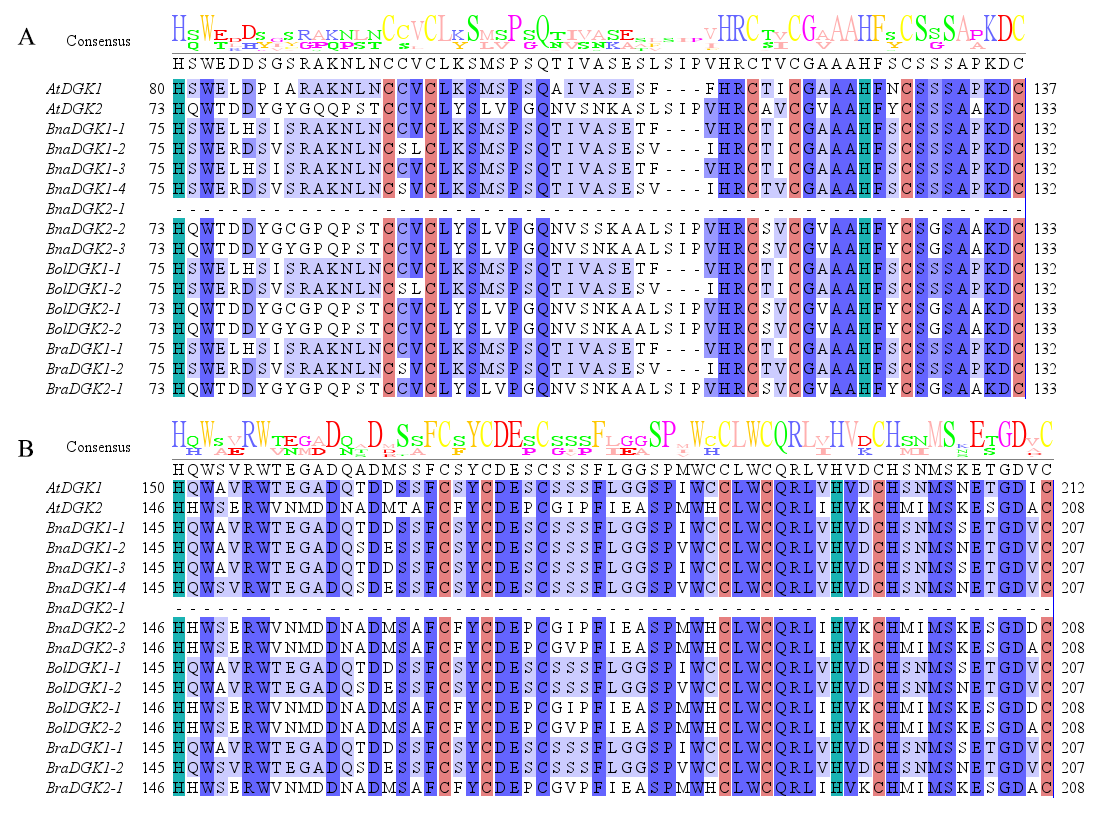

Supplement: Supplementary file 2 — Additional file 2: Figure S2. The multiple sequence alignment of two DAG/PE-binding domain (C1 domain) domains. (A) The first DAG/PE-binding domain, (B) The second DAG/PE-binding domain. [file 12870_2020_2691_MOESM2_ESM.tif]

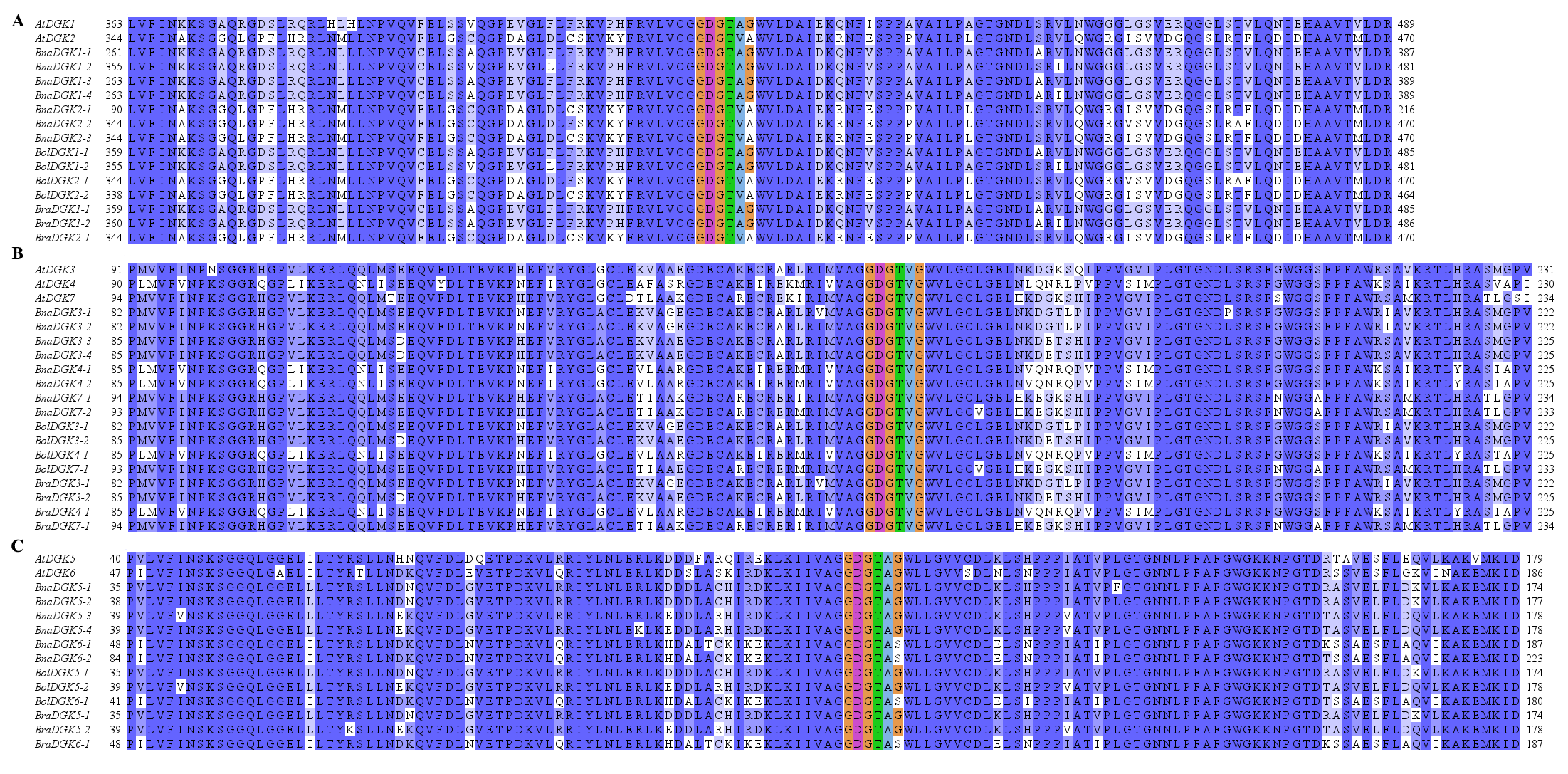

Supplement: Supplementary file 3 — Additional file 3: Figure S3. The multiple sequence alignment of each cluster DGKc domain among all DGK genes. [file 12870_2020_2691_MOESM3_ESM.tif]

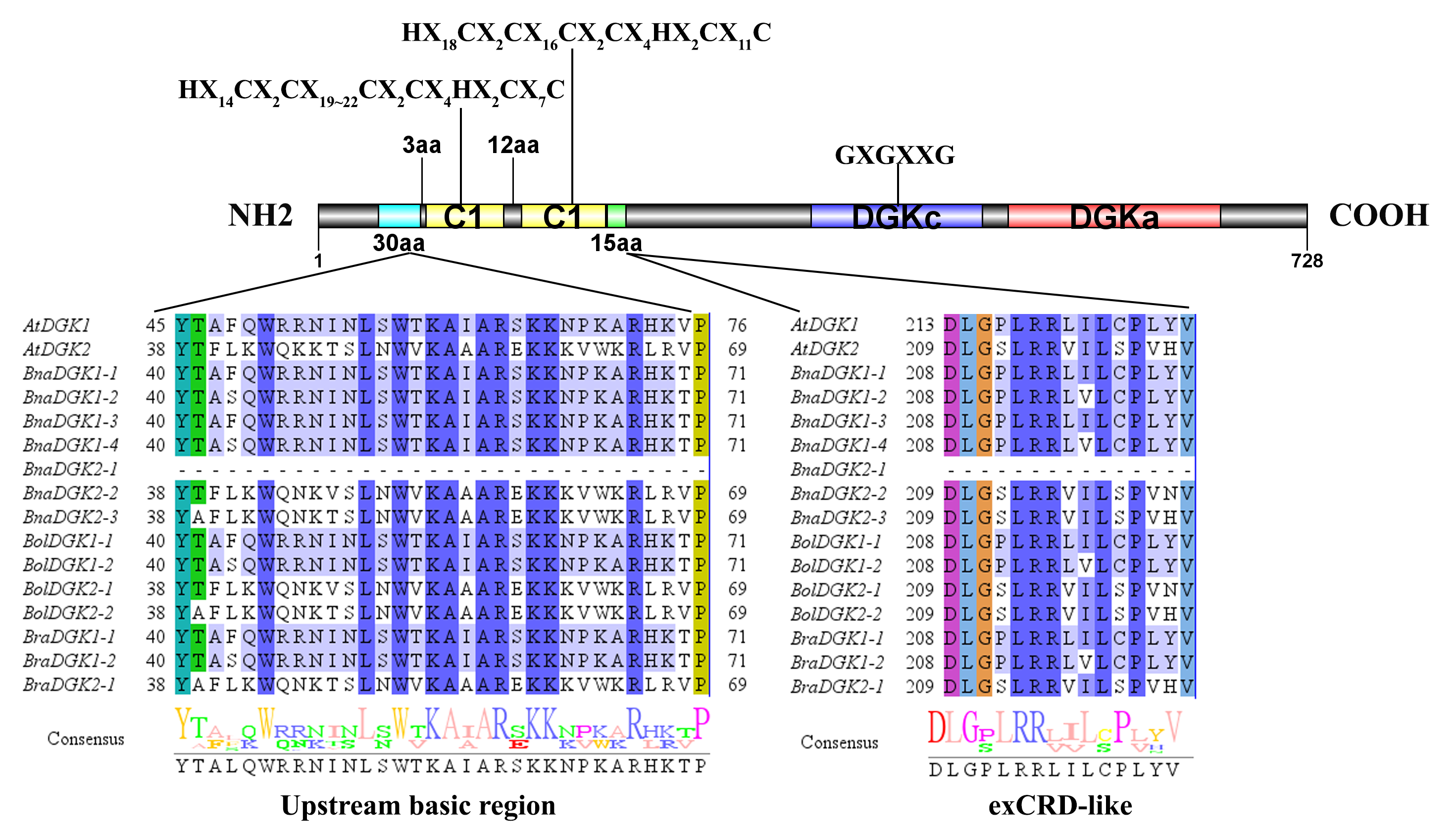

Supplement: Supplementary file 4 — Additional file 4: Figure S4. A detailed view of domains of DGK genes in cluster 1 including two C1 domains, the upstream basic regions and the extended cysteine-rich domain (extCRD). [file 12870_2020_2691_MOESM4_ESM.tif]

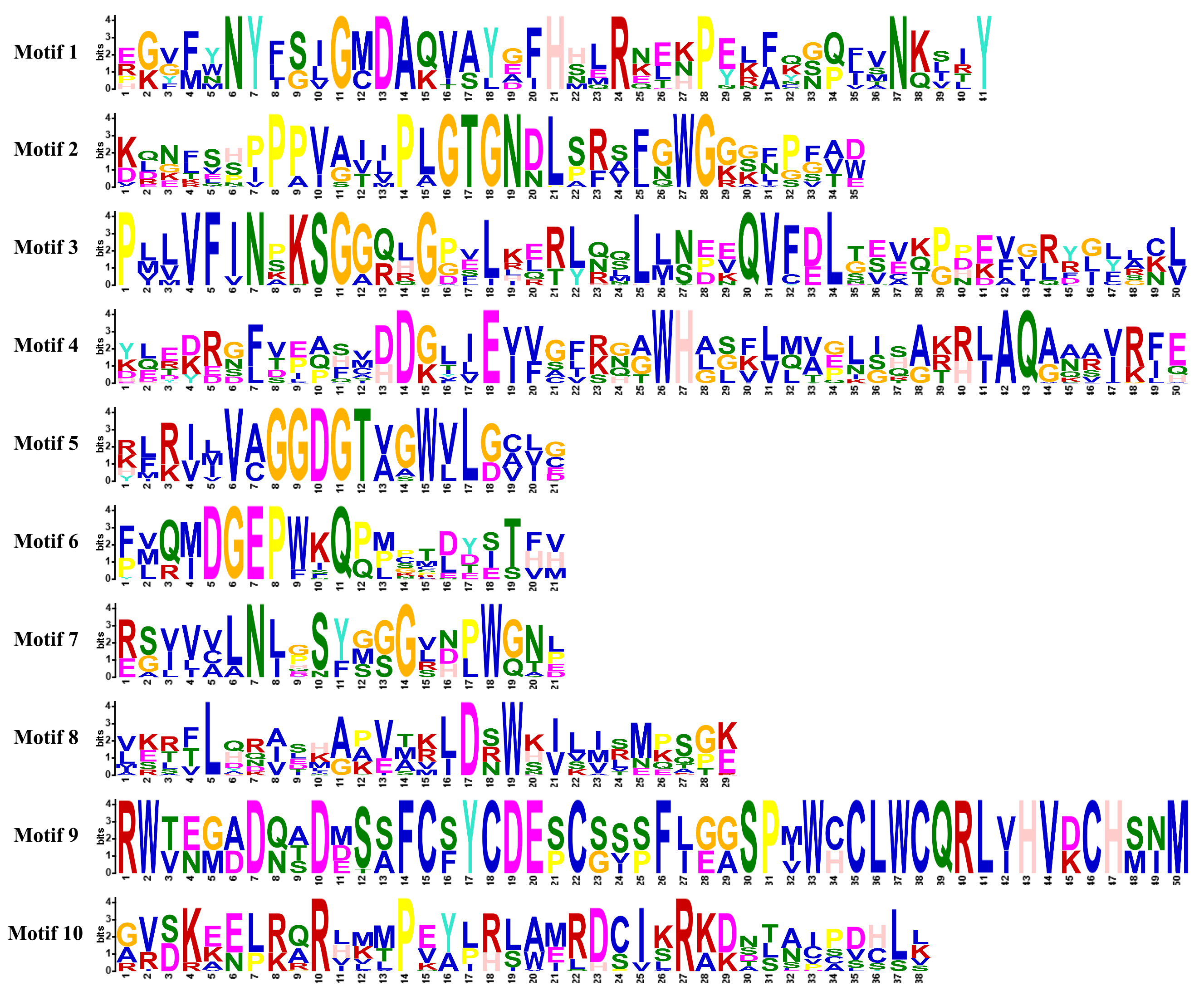

Supplement: Supplementary file 5 — Additional file 5: Figure S5. The detailed information of Motif logos are obtained from the MEME Suite website. [file 12870_2020_2691_MOESM5_ESM.tif]

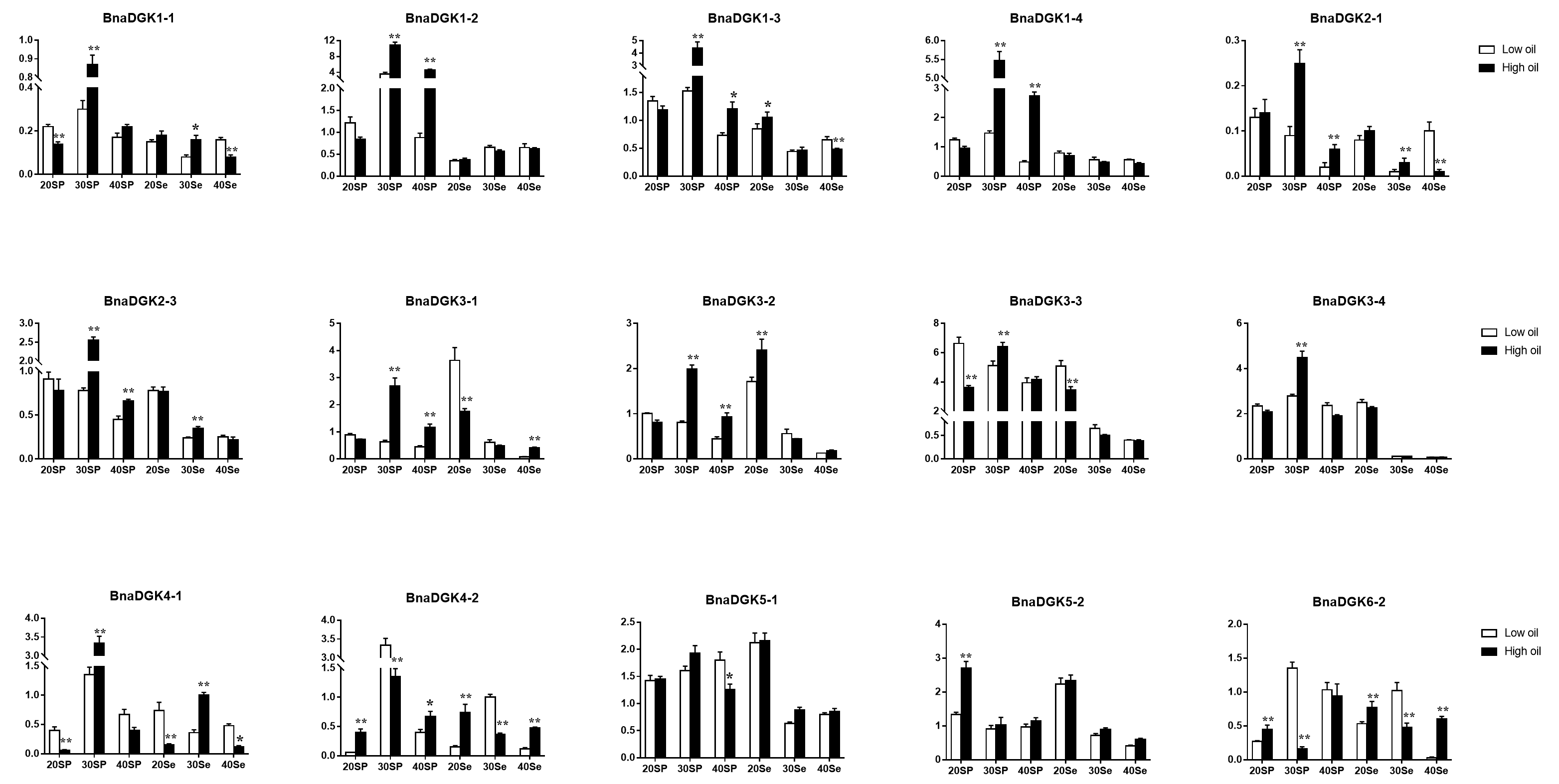

Supplement: Supplementary file 6 — Additional file 6: Figure S6. Quantitative RT-PCR analysis of the remaining BnaDGKs in different seed oil content materials. The expression levels of BnaDGKs were calculated using 2−ΔCt method. Bar values represent Means ± SEM of three biological replicates with three technical replicates. Asterisks indicate significant differences,* P < 0.05, ** P < 0.01. Se, Seed; SP, Silique pericarp. The number of days after flower (DAF) is indicated as 20, 30, 40d. [file 12870_2020_2691_MOESM6_ESM.tif]

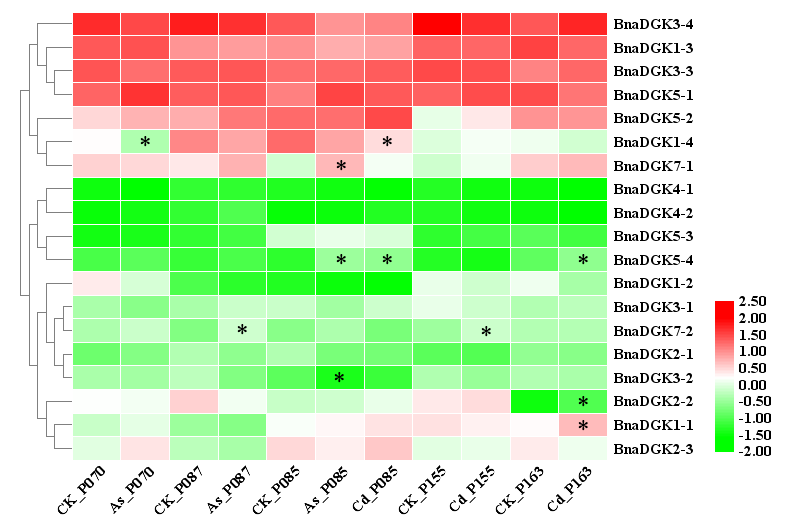

Supplement: Supplementary file 7 — Additional file 7: Figure S7. The heatmap of BnaDGKs expression profiling in response to As and Cd stress. The up-down regulation was defined with log2 ratio. [file 12870_2020_2691_MOESM7_ESM.tif]
